# The Diagnostic Yield of Abdominal Ultrasounds Requested by Family Physicians at an Academic Hospital in Riyadh, Saudi Arabia

**DOI:** 10.7759/cureus.25580

**Published:** 2022-06-01

**Authors:** Haytham I AlSaif, Abdullah S Alzaid, Mohammed A Albabtain, Abdulmalik A Alharbi, Fahad K Alnahabi, Ahmad I Alarifi, Mohammed S Alqasoumi, Abdulrahman Y Alhawas, Saad M Alsaad

**Affiliations:** 1 Department of Family and Community Medicine, College of Medicine, King Saud University, Riyadh, SAU; 2 College of Medicine, King Saud University, Riyadh, SAU; 3 Department of Radiology and Medical Imaging, College of Medicine, King Saud University, Riyadh, SAU

**Keywords:** saudi arabia, ultrasonography, abdomen, general practice, prevalence

## Abstract

Background: Abdominal ultrasound is a non-invasive, relatively inexpensive, and widely available diagnostic modality in family medicine settings.

Objectives: Our study aimed to identify the most common indications for requesting abdominal ultrasounds by family physicians, determine the frequency of abdominal ultrasound with abnormal findings, identify the most common findings, and determine patients’ characteristics associated with abnormal findings.

Methods: This retrospective chart-based study was conducted from January 2020 to June 2020 to analyze patients’ abdominal ultrasounds reports requested by family physicians in 2019 at King Khalid University Hospital (KKUH), Riyadh, Saudi Arabia.

Results: We assessed abdominal ultrasound reports of 1,113 patients. There were 620 (55.7%) female patients. The mean age and body mass index (BMI) were 46.35 years ± 15.04 and 29.33 kg/m^2^ ± 7.06, respectively. The most common indications were abdominal pain (43.2%), suspicion of gallbladder and biliary system diseases (18.5%), and abnormal liver function tests (14.6%). The frequency of abnormal findings was 793 (71.2%), and the most common findings were fatty liver infiltration (49.7%), liver enlargement (20.1%), and gallstones (13.3%). Females had a lower likelihood to have abnormal findings compared to males (odds ratio (OR)=0.688, p=0.009). Lastly, the likelihood of abnormal findings increased with age and was highest among patients aged 71 years or more (OR=25.9, p< 0.001).

Conclusion: Abnormal findings were more prevalent in our study compared with other studies. Abnormal findings were more common among males and older age groups. We recommend future studies on patients from community-based family medicine settings, and to examine the association of abnormal findings with patient-centered endpoints. Finally, disseminating the results of this study will inform family physicians with the most common abnormal abdominal ultrasound findings, and will enhance the discussion with patients undergoing an abdominal ultrasound examination.

## Introduction

Abdominal complaints are among the most common reasons for visiting a family physician [1]. They carry a broad differential diagnosis which can be narrowed with the aid of laboratory testing and imaging studies, particularly abdominal ultrasound [2]. Abdominal ultrasound is a non-invasive, relatively inexpensive, and widely available diagnostic modality in family medicine settings. The practice of utilizing ultrasound among family physicians has grown in the last two decades [3]. In the Netherlands, a high-income country with an estimated population of 17.4 million [4], general practitioners request yearly around 200,000 abdominal ultrasounds [2]. On average, a general practitioner would request around 131 to 601 ultrasound examinations per year, most of which (67%) are abdominal ultrasounds [5]. Abdominal ultrasound examinations are commonly requested for patients with abdominal pain in order to detect certain conditions, such as cholelithiasis, liver pathology, and nephrolithiasis [2].

Previous research showed that the frequency of abnormalities detected by abdominal ultrasound has ranged from 25.3% to 53.2% [2,6-9]. Gallstones (3.4%-19%) and fatty liver (0.5%-35.9%) were the most commonly reported abnormalities [2,6-11] . In addition, males had a higher frequency of abnormal findings [7,9]. In Saudi Arabia, previous reports among patients who underwent ultrasound examination have ranged between 3.7% and 16.6% for fatty liver [6,12] and between 6.6% and 11.3% for gallstones [6,13,14]. However, to the best of our knowledge, no studies in the literature reported differences in frequency of abnormal findings between genders in Saudi Arabia.

Our study aimed to identify the most common indications for requesting abdominal ultrasounds by family physicians, determine the frequency of abdominal ultrasound with abnormal findings, identify the most common findings, and determine patients’ characteristics associated with abnormal findings. As a result, this will improve the practices of family physicians and radiologists alike, inform the discussion with the patients about the yield of this imaging modality, and lead to optimal utilization of this service within the healthcare system.

## Materials and methods

Study design and setting 

This was a retrospective chart-review study. Data were abstracted from the electronic medical records from January 2020 to June 2020 at the King Khalid University Hospital (KKUH), a tertiary academic hospital in Riyadh, Saudi Arabia. The Department of Family Medicine at KKUH comprises approximately 60 clinics that served 124,349 patients during 2018. The minimum sample size needed to detect a proportion of 25.2% of abnormal findings with a 5% margin of error and 95% confidence level was 289 patients [6].

Study instrument 

A standardized data abstraction form of two parts was prepared. The first part included the following demographic variables: gender, age, and body mass index (BMI) from the electronic medical records. The second part included the following variables from the radiologist report: the indication for requesting the abdominal ultrasound, liver (size, presence of fatty changes, and presence of focal lesions), gallbladder (presence of stones, polyps, or cholecystectomy), kidneys (presence of stones, cysts, or masses, echogenicity, and hydronephrosis), and spleen size. Liver enlargement was defined as a liver span of more than 16 cm in the mid-clavicular line [15], while spleen enlargement was defined as a span of more than 12 cm in length [15]. 

Data collection 

A list of all abdominal ultrasound examinations done during 2019 was retrieved from the radiology department. Then the list was refined to include only orders by physicians from the family medicine department. Pediatric (less than 18 years old) and pregnant patients were excluded. In case more than one abdominal ultrasound examinations were done for the same patients, reports were combined in order to avoid counting the abnormality twice. Training of data abstractors (Abdullah S. Alzaid, Mohammed A. Albabtain, Abdulmalik A. Alharbi, Fahad K. Alnahabi, Ahmad I. Alarifi, and Mohammed S. Alqasoumi) was conducted by a consultant family physician (Haytham I. AlSaif). Then, a pilot study on 80 patients was performed during December 2019 to finalize the data abstraction form, address any potential issues, and unify the data abstraction process. Lastly, data were abstracted from the electronic medical records using the standardized data abstraction form.

Data management and statistical analysis 

Data were tabulated and managed using Excel version 16.0 (Microsoft, Redmond, USA) and analyzed using SPSS version 24.0 (IBM Corp, Armonk, USA). Descriptive statistics (means, medians, standard deviations, frequencies, and percentages) were used to describe the quantitative and categorical variables. Bivariate statistical analysis was carried out using Chi-square test of independence or independent sample t-test. A binary logistic regression model was run to see the effects of age, gender, and BMI on the likelihood of abnormal findings. Odds ratio (OR) and 95% confidence interval (CI) were used to report the results of logistic regression. A p-value of <0.05 was used to report the statistical significance.

## Results

Baseline demographic characteristics

A total of 1,161 patients underwent abdominal ultrasound examinations requested by family physicians in 2019. We excluded 48 patients from the study as they fulfilled the exclusion criteria, or no report was available in the medical records. Therefore, 1,113 patients were included in the analysis, for whom 1,134 abdominal ultrasound examinations were done. There were 17 patients (1.5%) and two patients (0.2%) who underwent abdominal ultrasound examination two and three times, respectively. More than half the patients (55.7%) were females. The median age was 45 years (interquartile range, 34.5-58 years). More than one-third of the patients (36.5%) were overweight (BMI=25-29.9 kg/m^2^), and mean BMI was 29.69±7.89 kg/m^2^ and 28.88±5.81 kg/m^2^ for females and males, respectively. Frequencies of demographic variables are shown in Table 1.

**Table 1:** Frequency of demographic characteristics of study participants (n=1113)

Variables	n	%
Gender		
Female	620	55.7
Male	493	44.3
Age (years)		
18-30	165	14.8
31-40	274	24.6
41-50	225	20.2
51-60	243	21.9
61-70	138	12.4
71 and above	68	6.1
Mean±SD	46.35 ± 15.04	
BMI (kg/m²)		
18.5 and below	25	2.2
18.6-24.9	233	20.9
25-29.9	406	36.5
30-34.9	265	23.8
35-39.9	104	9.3
40 and above	62	5.6
Not documented	18	1.62
Mean±SD	29.33 ± 7.06	

Indications and abnormal findings

**Figure 1:**
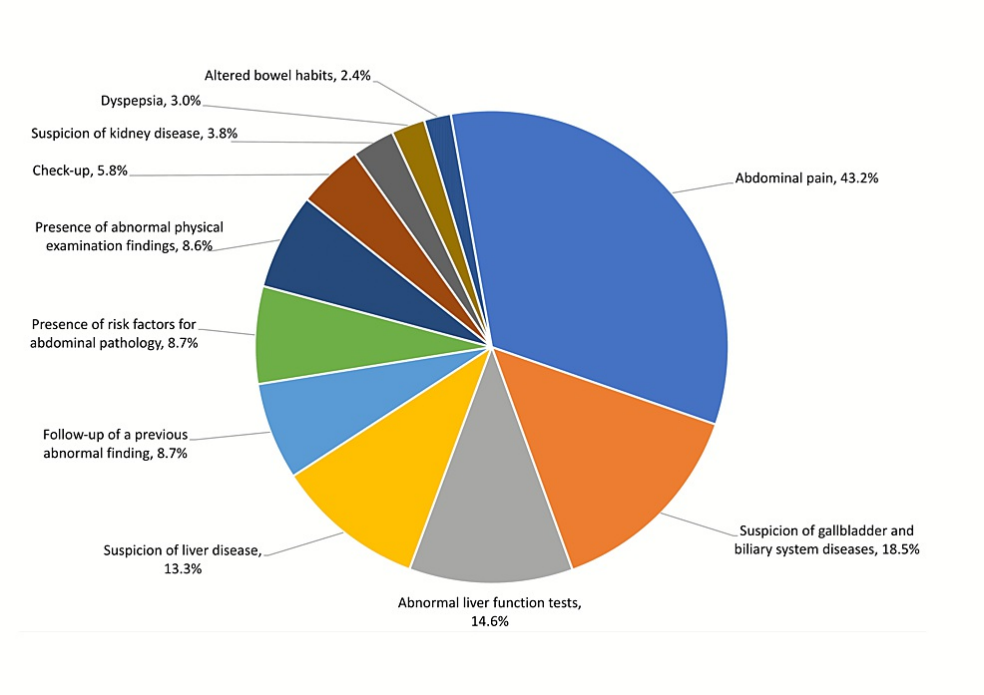
Indications for ordering abdominal ultrasound by family physicians

The most frequent indication was abdominal pain (43.2%), followed by suspicion of gallbladder and biliary system diseases (18.5%) and abnormal liver function tests (14.6%). Figure 1 shows the frequencies of different indications of abdominal ultrasound. The majority of abdominal ultrasound examinations (793, 71.2%) reported abnormal findings, and the most abnormal findings were located in the liver (57.8%), gallbladder (23.1%), right kidney (13.1%), and left kidney (12.2%). The most common abnormal findings were fatty liver infiltration (49.7%), liver enlargement (20.1%), gallstones (13.3%), and renal cysts (9.1%). More renal cysts were detected in the right kidney (n = 66; 5.9%) compared with the left kidney (n = 51; 4.6%). The following abnormalities were significantly more common among females: gallstones, focal liver lesions, and renal masses. However, fatty liver and renal cysts were more common among males. The mean age of patients was significantly higher for the following abnormalities: fatty liver, liver enlargement, focal liver lesion, gallstone, cholecystectomy, renal cyst, renal hyperechogenecity, and hepatic cystic lesion. Patients with renal hydronephrosis had significantly lower BMI. The frequencies of abnormal findings and its relation to demographic characteristics are shown in Table 2.

**Table 2:** Frequencies and associations of abnormal findings with demographic characteristics *  Chi-square test of independence. ** Independent-samples t-test.

Findings			Gender		Age	BMI
		n (%)		n (%)	Mean ± SD	Mean ± SD
				P-Value*	P-Value**	P-Value**
Fatty Liver	Yes	553 (49.7)	Female	261 (47.2)	49.26 ± 13.68	29.26 ± 5.98
	No	560 (50.3)	Male	292 (52.8)	43.48 ± 15.69	29.40 ± 7.98
				<0.001	<0.001	0.739
Liver Enlargement	Yes	224 (20.1)	Female	131 (58.5)	48.3 ± 13.01	29.5 ± 5.38
	No	889 (79.9)	Male	93 (41.5)	45.65 ± 15.41	29.3 ± 7.37
				0.349	0.001	0.766
Focal Liver Lesion	Yes	71 (6.4)	Female	50 (70.4)	49.48 ± 12.68	30.89 ± 16.92
	No	1024 (93.6)	Male	21 (29.6)	46.16 ± 15.15	29.22 ± 6.13
				0.010	0.047	0.355
Spleen Enlargement	Yes	24 (2.2)	Female	10 (41.7)	35.82 ± 13.22	28.65 ± 4.87
	No	1089 (97.8)	Male	14 (58.3)	46.43 ± 15.02	29.31 ± 7.09
				0.162	0.245	0.405
Gallstone	Yes	148 (13.3)	Female	96 (64.9)	50.20 ± 14.33	29.83 ± 5.84
	No	965 (86.7)	Male	52 (35.1)	45.76 ± 15.02	29.25 ± 7.22
				0.016	0.001	0.356
Gallbladder Polyp	Yes	40 (3.6)	Female	22 (55.0)	46.28 ± 11.80	31.26 ± 19.47
	No	1073 (96.4)	Male	18 (45.0)	46.36 ± 15.11	29.26 ± 6.12
				0.927	0.966	0.52
Cholecystectomy	Yes	67 (6)	Female	57 (85.1)	55.57 ± 14.08	29.23 ± 5.58
	No	1046 (94)	Male	10 (14.9)	45.76 ± 14.87	29.34 ± 7.14
				<0.001	<0.001	0.902
Renal Stone	Yes	23 (2.1)	Female	13 (56.5)	50.13 ± 14.47	27.57 ± 6.2
	No	1090 (97.9)	Male	10 (43.5)	46.27 ± 15.01	29.36 ± 7.07
				0.937	0.223	0.226
Renal Cyst	Yes	101 (9.1)	Female	45 (44.6)	60.34± 13.9	29.39± 6.35
	No	1012 (90.9)	Male	56 (55.4)	44.96 ± 14.39	29.33 ± 7.12
				0.018	<0.001	0.934
Renal Mass	Yes	21 (1.9)	Female	18 (85.7)	57.70 ± 12.94	27.97 ± 5.91
	No	1092 (98.1)	Male	3 (14.3)	46.14 ± 14.97	29.36 ± 7.07
				0.005	<0.001	0.3
Hydronephrosis	Yes	20 (1.8)	Female	14 (70)	47.05 ± 14.9	27.83± 3.06
	No	1093 (98.2)	Male	6 (30)	46.34 ± 15.01	29.36 ± 7.11
				0.194	0.834	0.044
Renal Hyperechogenicity	Yes	34 (3.1)	Female	18 (52.9)	68.12 ± 13.95	27.95 ± 5.11
	No	1079 (96.9)	Male	16 (47.1)	45.67 ± 14.52	29.37 ± 7.1
				0.742	<0.001	0.252
Hepatic Hemangioma	Yes	31 (2.8)	Female	28 (90.3)	44.04 ± 9.94	33.27 ± 21.45
	No	1082 (97.2)	Male	3 (9.7)	46.42 ± 15.12	29.22 ± 6.15
				<0.001	0.2	0.305
Hepatic Focal Fatty Sparing	Yes	17 (1.5)	Female	8 (47.1)	48.59 ± 11.68	30.92 ± 6.63
	No	1096 (98.5)	Male	9 (52.9)	46.32 ± 15.05	29.30 ± 7.06
				0.470	0.536	0.35
Hepatic Cystic Lesion	Yes	12 (1.1)	Female	7 (58.3)	61.03 ± 12.07	27.77 ± 7.11
	No	1101 (98.9)	Male	5 (41.7)	46.19 ± 14.96	29.34 ± 7.05
				0.854	0.001	0.442

Association between abnormal findings and demographic variables

Abnormal findings were more common among males and older age groups. However, there was no significant difference in frequency across BMI groups. Table 3 shows the frequency of abnormal findings across the categories of demographic variables. Adjusted associations between demographic variables and abnormal findings were assessed using binary logistic regression. Females showed a lower likelihood of having abnormal findings compared to males (OR=0.688, p=0.009). Furthermore, the likelihood of abnormal findings increased with age and was highest among patients aged 71 years or more (OR=25.9, p< 0.001). However, BMI did not exhibit a statistically significant association with abnormal findings (p=0.565). Results of binary logistic regression are shown in Table 4.

**Table 3:** Differences in the distribution of abnormal findings across demographic characteristics (n=1113) * Chi-square test of independence. ** 18 patients out of 1113 did not have BMI documentation.

Variables		Abnormal Findings n (%)		Total	P-Value*
		Yes	No		
Gender	Female	423 (68.2)	197 (31.8)	620	0.012
	Male	370 (75.1)	123 (24.9)	493	
	Total	793 (71.2)	320 (28.8)	1113	
Age (years)	18-30	64 (38.8)	101 (61.2)	165	<0.0001
	31-40	177 (64.6)	97 (35.4)	274	
	41-50	176 (78.2)	49 (21.8)	225	
	51-60	197 (81.1)	46 (18.9)	243	
	61-70	115 (83.3)	23 (16.7)	138	
	71 and above	64 (94.1)	4 (5.9)	68	
	Total	793 (71.2)	320 (28.8)	1113	
BMI (kg/m²)	18.5 and below	15 (60)	10 (40)	25	0.679
	18.6-24.9	162 (69.5)	71 (30.5)	233	
	25-29.9	289 (71.2)	117 (28.8)	406	
	30-34.9	188 (70.9)	77 (29.1)	265	
	35-39.9	75 (72.1)	29 (27.9)	104	
	40 and above	48 (77.4)	14 (22.6)	62	
	Total	777 (71)	318 (29)	1095**	

**Table 4:** Demographic characteristics associated with abnormal findings: binary logistic regression results CI = confidence interval; OR = odds ratio; SE = standard error.

Variables	β	SE	P-Value	OR	95% CI for OR	
Gender (reference: male)						
Female	-0.359	0.146	0.014	0.698	0.525	0.929
Age group (reference: 18-30 years)						
31-40	1.065	0.209	<0.001	2.901	1.927	4.368
41-50	1.77	0.232	<0.001	5.869	3.727	9.242
51-60	1.968	0.234	<0.001	7.158	4.522	11.33
61-70	2.073	0.284	<0.001	7.945	4.556	13.856
71 years ≤	3.252	0.543	<0.001	25.854	8.926	74.888
BMI (reference: : 18.6-24.9 kg/m²)						
≤ 18.5 kg/m²	-0.041	0.473	0.931	0.96	0.379	2.427
25-29.9 kg/m²	0.187	0.193	0.331	1.206	0.827	1.759
30-34.9 kg/m²	0.02	0.21	0.923	1.02	0.677	1.539
35-39.9 kg/m²	0.28	0.282	0.32	1.323	0.762	2.299
40 kg/m² ≤	0.326	0.351	0.353	1.385	0.697	2.754
Constant	-0.409	0.231	0.077	0.664		

## Discussion

The most common two indications for ordering ultrasound in our study were abdominal pain and suspicion of gallbladder disease. This is consistent with what was reported by Speets et al. [2]. Interestingly, the third most common indication in our study was a further investigation following abnormal liver function tests. The American College of Gastroenterology (ACG) recommends repeating abnormal liver chemistries before initiating the evaluation of abnormal tests [16]. Family physicians should follow this recommendation in order to achieve a wiser utilization of abdominal ultrasound. Utility of abdominal ultrasound for check-up or screening is only recommended for patients at higher risk of abdominal aortic aneurysm [17]. Lastly, abdominal ultrasonography is not typically recommended for patients presenting with dyspepsia unless there is a presence of red flags [18].

Abdominal ultrasound examinations in our study had higher abnormal findings (71.2%) compared with previous research (25.3%-53.2%) [2,6-9]. However, in our study, we did not exclude abdominal ultrasound examinations requested for a follow-up of a previous abnormal finding (8.7%). Yet even if they were excluded, the frequency of abnormal findings would be at least 68.5%, which is still higher that what was previously reported. This difference can be explained by the following factors: first, the high mean BMI (29.33 kg/m²) in our sample, since it is a known risk factor for fatty liver [19], which was the most frequent abnormal finding in our study (49.7%). Second, our sample, which was drawn from family medicine clinics that are part of a tertiary center, might have included more patients with chronic conditions such as diabetes mellitus and hypertension than community-based family medicine clinics. The proportion of abnormal findings was higher in males, which goes along with previous research [7,9]. Nevertheless, more female patients were referred for an abdominal ultrasound. This can be attributed to the fact that female patients utilize health care services more than their male counterparts [20-22].

The most common finding in our study was fatty liver infiltration (49.7%), which is higher than what was reported previously in the literature (0.5%-35.9%) [2,6-11]. A previous study conducted in 2009 at the same center showed a much lower prevalence of fatty liver (16.6%) [12]. Contrary to our study, patients with existing liver disease or alcohol use were excluded, and all ultrasounds were read by one radiologist [12]. Despite these differences, we believe that the findings from our study might reflect a rising trend in non-alcoholic fatty liver disease (NAFLD) in Saudi Arabia, which was suggested previously based on a modeling study that utilized obesity and diabetes mellitus prevalence data [23]. This calls for more action from family physicians to control the risk factors for this condition and detect it early among their patients. Interestingly, despite obesity being a risk factors for fatty liver [17], the mean BMI for patients with fatty liver was not significantly higher than those without. Lastly, fatty liver is one of the causes of liver enlargement, which represented the second most common finding in our study [24].

The frequency of gallstones in our study was 13.3% which falls within the range of previous studies (3.4%-19%) [2,6-11]. Previous Saudi community-based studies revealed a slightly lower (8.6% and 11.7%) prevalence of gallstones compared with our study [13]. Moreover, 6% of patients in our study previously underwent cholecystectomy, this is consistent with Speets et al. where 7% of patients in their study underwent cholecystectomy. In addition, 3.6% of patients had gallbladder polyps, which is lower than the prevalence reported in the literature (6.1%-7.4%) [25-27]. Lastly, renal cysts were the most common abnormality detected in the kidneys in our study (9.1%), which is within the range of what was reported previously in other studies that used ultrasound (3.1%-14%) [28]. Consistent with the literature, being male and older in age were associated with renal cysts in our study [28-30].

Strengths and limitations

This study included a relatively large sample size attending an academic family practice. To the best of our knowledge, this is the first report from Saudi Arabia that investigates specifically abdominal ultrasound findings ordered by family physicians. However, this study was limited by its retrospective design, including patients from a single center, and collecting limited patients’ characteristics. Lastly, imaging and reporting were conducted by various technicians and radiologists, which might be a potential source of variability in reporting.

Recommendations

Family physicians should follow the ACG recommendation of repeating abnormal liver chemistries before proceeding to further investigation, such as abdominal ultrasound. Moreover, more efforts are needed to control the risk factors for the rising trend of fatty liver disease. Further studies should be done on patients from community-based family medicine practice. Also, studies examining in details the predictors and distribution of specific abnormalities are needed. Lastly, disseminating the results of this study to improve the practice of family physicians and radiologists, and to inform the discussion with patients undergoing abdominal ultrasound examinations.

## Conclusions

The most common indications for ordering abdominal ultrasound were abdominal pain, suspicion of gallbladder and biliary system diseases, and abnormal liver function tests. We found higher frequency of abnormal findings compared to the previous research, possibly because of higher frequency of fatty liver disease. However, the frequencies of other abnormal findings were consistent with the literature. Lastly, older age and male gender were significantly associated with abnormal findings.
